# 1例血友病A合并高滴度抑制物患者胸腔镜肺叶切除术的围手术期止血管理及文献复习

**DOI:** 10.3760/cma.j.cn121090-20250113-00026

**Published:** 2025-08

**Authors:** 芝君 孟, 捷 项, 冠群 许, 禹 刘, 秋兰 丁, 菁 戴, 文漫 武, 学锋 王

**Affiliations:** 1 山西省人民医院检验科，山西 030012 Department of Laboratory Medicine, Shanxi Provincial People's Hospital, Taiyuan 030012, China; 2 上海交通大学医学院附属瑞金医院胸外科，上海 200025 Department of Thoracic Surgery, Ruijin Hospital, Shanghai Jiao Tong University School of Medicine, Shanghai 200025, China; 3 上海交通大学医学院附属瑞金医院检验科，上海 200025 Department of Laboratory Medicine, Ruijin Hospital, Shanghai Jiao Tong University School of Medicine, Shanghai 200025, China

## Abstract

1例64岁男性血友病A患者，因“肺占位”拟行手术治疗。术前发现凝血因子Ⅷ（FⅧ）活性0.5％、FⅧ抑制物32 BU/ml，血栓弹力图R值无法检测。顺利完成胸腔镜肺叶切除术，手术当天及术后第1天予以活化凝血因子Ⅶ（rFⅦa）6 mg每6 h 1次静脉滴注。术后第1天发现血压下降、HGB由102 g/L逐渐降至65 g/L，X线胸片显示左侧大量胸腔积液；急行胸腔镜下胸腔探查，清理新鲜积血3 200 ml并放置胸腔引流管。第2次术后，rFⅦa加量至6 mg每4 h 1次静脉滴注，间断输注去白红细胞纠正贫血。4 d后因rFⅦa不能获得，改用凝血酶原复合物50 IU/kg每8 h 1次，同时予以清除FⅧ抑制物治疗（甲泼尼龙40 mg/d、环磷酰胺200 mg每周2次）。术后第9天拔除胸腔引流管，术后3周痊愈出院。

## Abstract (en)

A 64-year-old male patient with hemophilia A was scheduled for the surgical removal of a pulmonary mass. Preoperative evaluation revealed that the coagulation factor Ⅷ（FⅧ）activity was 0.5％, with an FⅧ inhibitor level of 32 BU/ml; the R value could not be detected on the thromboelastogram. Thoracoscopic lobectomy was successfully completed. On the day of the operation and the first day after the operation, 6 mg of recombinant activated coagulation factor Ⅶ（rFⅦa）was intravenously administered every 6 h. On postoperative day 1, the patient's blood pressure dropped and the HGB gradually declined from 102 g/L to 65 g/L. Chest X-ray revealed a large amount of pleural effusion on the left side, and urgent thoracoscopic thoracic exploration was performed. A total of 3200 mL fresh blood was cleared, and a thoracic drainage tube was placed. On postoperative day 2, the rFⅦa dose was increased to 6 mg, which was intravenously administered every 4 h, and concentrated red cells were intermittently infused to correct anemia. Four days later, due to the inability to obtain rFⅦa, PCC（50 IU/kg every 8 hours）was administered. Additionally, treatment with methylprednisolone（40 mg/d）and cyclophosphamide（200 mg, every 2 weeks）was initiated to remove FⅧ inhibitors. The thoracic drainage tube was removed on postoperative day 9, and the patient was successfully discharged 3 weeks later.

血友病A是最常见的X染色体连锁隐性遗传的出血性疾病，是凝血因子Ⅷ（FⅧ）的质或量异常引起轻微损伤或自发性关节、内脏和肌肉出血，可导致患者关节活动障碍并致残。我国血友病患病率为（2.73～3.09）/10万[1]。在接受外源性FⅧ治疗后，约1/3的重型血友病A患者会产生抑制物[2]，是血友病A治疗过程中最棘手的并发症。我们应用活化凝血因子Ⅶ（rFⅦa）和凝血酶原复合物（PCC）旁路药物在1例合并高滴度抑制物血友病A患者肺癌手术围手术期进行止血管理并获得成功，报告如下并复习相关文献。

## 病例资料

患者，男性，64岁，既往诊断血友病A，2024年3月CT检查显示左肺下叶胸膜下见团块状软组织影（37 mm×31 mm），门诊拟诊断为“肺占位”，于2024年4月入住上海瑞金医院拟行手术治疗。入院后检查活化部分凝血活酶时间（APTT）：96.1 s（参考值33 s），APTT纠正实验：正浆与病浆1∶1混合后即刻40.9 s，37 °C孵育2 h后72.1 s。FⅧ活性0.5％，FⅧ抑制物32 BU/ml。血栓弹力图R值无法检测。多学科会诊后设定手术止血方案：手术当天和术后第1天rFⅦa 6 mg每6 h 1次静脉滴注，术后第2～4天rFⅦa 4 mg每6 h 1次。胸腔镜经左前外侧第五肋间进胸，探查见左下肺一质感硬肿物，大小约4 cm×5 cm，完整切除左肺下叶，清扫5、6、7、10、11组淋巴结。术中输注冷沉淀2 U、去白红细胞1 U。手术过程顺利，术后切口无渗血渗液。术后第1天患者出现血压下降、呼吸急促，HGB由102 g/L逐渐降至65 g/L，胸片显示左侧大量胸腔积液；急行胸腔镜下胸腔探查，发现左侧胸腔内鲜红色血液及血块3200 ml，吸尽出血及胸腔冲洗后，肺门及切口渗血处严密止血并放置胸腔引流管；输注去白红细胞8 U，rFⅦa改为6 mg每4 h 1次静脉滴注。术后第2天（第2次术后第1天），胸腔引流约60 ml血性液体，HGB 114 g/L，持续rFⅦa 6 mg每4 h 1次静脉滴注，输注去白红细胞8 U；术后第3天，胸腔引流约50 ml血性液体，HGB 54 g/L，输注去白红细胞2 U；术后第4天，胸腔引流30 ml血性液体，HGB 46 g/L，血小板计数94×10^9^/L，输注去白红细胞6 U、去白单采血小板1 U，无异常活动性出血，止血方案更改为PCC 50 U/kg，每8 h 1次（因rFⅦa短缺）并加用甲泼尼龙40 mg/d；术后第5天，胸腔引流约20 ml血性液体，HGB 78 g/L，输注去白红细胞2 U，加用环磷酰胺200 mg每周2次以清除抑制物；术后第6～7天，胸腔引流约5 ml血性液体，HGB 86 g/L；第2次术后第8天，胸腔引流约3 ml血性液体，患者停止镇静镇痛，予以停用呼吸机并拔除引流管。术后3周，手术创口愈合，痊愈出院。病理结果：鳞状细胞癌，角化型，组织学分化为中分化。围手术期血栓弹力图和相关凝血指标检测结果见表1。

**表1:** 一例血友病A合并高滴度抑制物患者肺叶切除术围手术期凝血相关监测指标

检测项目	术前	术后									
		0 d	1 d	2 d	3 d	4 d	5 d	7 d	11 d	14 d	19 d
APTT（s）	96.1	88.9	88.3	88.2	114.4	107.6	105.3	105.7	101.2	120.0	115.6
PT（s）	11.3	7.4	7.8	7.7	7.7	7.7	11.5	12.2	13.0	15.4	13.5
D-D（mg/L）	0.9	1.27	0.65	0.33	0.37	3.10	5.26	8.18	7.62	6.42	5.26
TEG											
R时间（min）	/	/	20.42	12.7	15.8	32.0	26.6	42.6	46.00	34.30	25.20
K时间（min）	/	/	3.96	3.2	5.2	6.20	6.3	12.5	9.10	11.20	9.10
α角	/	/	42.87	49.0	35.4	34.30	33.3	18.9	27.60	22.70	26.70
综合凝血指数	/	/	−11.92	−6.40	−10.0	−20.20	−17.5	−31.3	−29.60	−24.10	−16.40
HGB（g/L）	125	82	65	114	54	46	78	86	85	80	78
FⅧ活性（％）	0.5	/	/	/	/	/	0.5	0.5	/	/	0.5
FⅧ抑制物（BU）	32	/	/	/	/	/	16	8	/	/	4

**注** APTT：活化部分凝血活酶时间；PT：凝血酶原时间；D-D：D-二聚体；TEG：血栓弹力图；FⅧ：凝血因子Ⅷ；/：无数据

## 讨论

对体内存在高滴度抑制物（>5 BU/ml）的成人血友病A患者，国内指南推荐清除抑制物或将抑制物滴度降至低滴度水平（<5 BU/ml）后再行择期手术[3]。本例患者临床高度怀疑肺癌，没有充足的时间进行清除抑制物的治疗。值得注意的是，患者在术后止血情况相对稳定后，我们采取了止血和清除抑制物同时进行的治疗方式，其抗体滴度从32 BU/ml降至4 BU/ml。

对于血友病A合并高滴度抑制物（≥5 BU/ml）的患者，有创治疗最常用的止血方案为“旁路途径”药物[4]。目前可供选择的“旁路途径”药物包括基因重组的rFⅦa及PCC[6]。本案例中，采用rFⅦa 6 mg（90 µg/kg）每6 h 1次静脉输注，持续48 h。由于术后第1天发生胸腔大量血性积液，再次进行手术探查止血，两次手术后rFⅦa 6 mg（90 µg/kg）更改为每4 h静脉输注1次，持续48 h。由于rFⅦa短缺，后续更改为PCC进行止血治疗。文献报道，在治疗关节出血时，2剂rFⅦa 90～120 µg/kg与1剂aPCC 75～100 IU/kg疗效大致相当。指南推荐PCC的用法为50～100 IU/kg每8～12 h 1次静脉给药，一天总量不超过150 IU/kg[3]。本例患者采用PCC（50 U/kg）每8 h 1次，至出院并未发生血栓。而在既往报告的一例血友病A合并抑制物（2.6 BU/ml）进行左侧跟腱延长术、肌腱吻合术的案例中采用了rFⅦa联合PCC的序贯联合旁路治疗（SCBT）方法进行围手术期止血管理，在术后第13天发生了下肢静脉血栓[6]。因此，在PCC使用的过程中需要警惕发生血栓事件并尽量避免与抗纤溶药物同时使用（如必须使用，则需要间隔6 h以上）[3]。本例患者为合并高滴度抑制物血友病A的老年男性，由于客观原因，围手术期采用了单一的旁路治疗（同时增加抑制物清除治疗）方案，也获得了较为满意的止血效果。

血友病A合并抑制物患者在进行手术治疗时，围手术期对患者体内的凝血状态进行监测是极其重要的。然而，常规的凝血检测项目（APTT、PT、抗凝血酶、D-二聚体及FⅧ活性）往往不能精确评估采用“旁路途径”药物治疗患者的体内凝血状态。有学者比较血栓弹性测定和凝血酶生成试验（TGA）后发现二者均可在伴抑制物血友病患者中整体评估旁路治疗后患者体内的凝血状态[7]。我们应用血栓弹力图对本例患者的凝血状态进行评估，观察到rFⅦa或PCC输注后R时间出现缩短，综合凝血指数也呈现相应的变化趋势，期间患者未再发生出血。患者住院期间APTT基本保持在100 s左右，对评估患者凝血功能的作用欠佳。

综上，我们成功应用rFⅦa和PCC旁路药物为一例合并高滴度抑制物血友病A患者进行肺癌手术围手术期止血管理，可为今后血友病A合并高滴度抑制物患者的围手术期止血处置提供参考。
